# Vital-Signs Detector Based on Frequency-Shift Keying Radar

**DOI:** 10.3390/s20195516

**Published:** 2020-09-26

**Authors:** Jae Young Sim, Jae-Hyun Park, Jong-Ryul Yang

**Affiliations:** Department of Electronic Engineering, Yeungnam University, Gyeongsan, Gyeongbuk 38541, Korea; ja2922@yu.ac.kr (J.Y.S.); bravopark@ynu.ac.kr (J.-H.P.)

**Keywords:** frequency-shift keying radar, cross-correlation, envelope detection, continuous-wave radar, frequency discrimination, vital-signs monitoring, heartbeat accuracy improvement, heartbeat detection, absolute distance measurement, radar signal processing

## Abstract

A frequency-shift keying (FSK) radar in the 2.45-GHz band is proposed for highly accurate vital-signs detection. The measurement accuracy of the proposed detector for the heartbeat is increased by using the cross-correlation between the phase differences of signals at two frequencies used by the FSK radar, which alternately transmits and receives the signals with different frequencies. Two frequencies—2.45 and 2.5 GHz—are effectively discriminated by using the envelope detection with the frequency control signal of the signal generator in the output waveform of the FSK radar. The phase difference between transmitted and received signals at each frequency is determined after calibrating the *I*/*Q* imbalance and direct-current offset using a data-based imbalance compensation algorithm, the Gram–Schmidt procedure, and the Pratt method. The absolute-distance measurement results for a human being show that the vital signs obtained at each frequency using the proposed FSK radar have a cross-correlation. The heartbeat detection results for the proposed FSK radar at a distance of < 2.4 m indicate a reduction in the error rate and an increase in the signal-to-noise ratio compared with those obtained using a single operating frequency.

## 1. Introduction

Research on vital-signs detection using radar technology has been performed since the short-range radar system was introduced for the detection of human vital signs in the 1970s [1]. A vital-signs detector based on radar technology can measure the vital signs without electrode contacts or restriction of the measurement environment, in contrast to contact-type detectors [2]. Owing to these advantages, the radar sensor for vital-signs detection is promising as a key element in continuous monitoring systems for home-care service, local positioning and tracking in disaster scenes such as earthquakes and fires, as well as disease identification using heartrate variability (HRV) analysis, e.g., sleep apnea and angina pectoris [3,4,5]. In particular, the study on a vital-signs detector using radar technology aims to achieve a level of detection accuracy so as to fully replace the electrocardiogram (ECG) sensor in medical applications, such as HRV analysis.

Among the radar technologies, continuous-wave (CW) Doppler radars are useful for vital-signs monitoring based on periodic motions in the human body because of their simple hardware configuration and signal processing [3,4,5,6]. However, CW radars have a limitation in that noise can be increased or the receiver can be saturated by the movement of the target or surrounding clutters, as signals caused by all movements are collected by the antenna [7]. This limitation causes particularly significant degradation of accuracy in heartbeat detection compared with respiration detection, because the chest movement caused by the heartbeat is 0.2–0.5 mm, whereas the chest movement caused by respiration is 4–12 mm [8,9]. It is important to implement the CW radar with a high signal-to-noise ratio (SNR) for heartbeat detection to increase the detection accuracy [10]. The SNR in the CW radar can be improved by increasing the transmitted power, but the maximum allowable effective isotropic radiated power is restricted by the regulation in each frequency band. It is not easy to implement a radar front-end with a high SNR by using a low-noise and high-gain design methodology, and a complex radar architecture including calibration circuits and a calibration process could be needed to improve the SNR [10]. The accuracy of vital-signs detection can be also improved by using signal-processing techniques such as autocorrelation, the wavelet transform, and cross-correlation [11,12,13,14]. The autocorrelation method improves the detection accuracy for the periodic signal by converging signals to the most representative period frequency but has limitations for accurately monitoring heartbeat signals that change over time and evaluating their variability [11,14]. The wavelet transform is useful for increasing the accuracy by effectively extracting peaks of the heartbeat signal, but it is difficult to develop a generalized wavelet function that can improve the accuracy while being independent of the measurement environment, such as the characteristics of the subject and the clutter in the surroundings [12,14]. The cross-correlation method was proposed for increasing the detection accuracy by exploiting the similarity between vital signs independently measured at multiple frequencies by using a dual-band antenna and commercialized measurement equipment [13]. Although this approach is useful for achieving a high accuracy because the vital signs are independent of the characteristics of the radar and the operation frequency, it is necessary to use several radars with different operating frequencies, and customized components such as a dual-band antenna are required.

In this work, a vital-signs detector with improved accuracy based on frequency-shift keying (FSK) radar technology is proposed. An FSK radar is used for a highly accurate range detection based on the phase difference between the transmitting and receiving signals separately obtained at more than two operating frequencies [15]. The vital-signs detection in the FSK radar is performed by using the same method as the CW Doppler radar as the FSK radar can be regarded as operating with several CW signals in the same hardware configuration. The proposed FSK radar improves the detection accuracy and SNR by using the cross-correlation between the vital signs independently obtained from each operating frequency. A method of effectively discriminating two frequency signals at the baseband output is necessary for accurately obtaining the results of the cross-correlation in the FSK radar [16]. This work presents the signal discrimination technique based on envelope detection in synchronization with a frequency control signal as a method for separately obtaining each operating frequency signal. The proposed technique can discriminate the phase information at each operating frequency from the FSK radar in a short period, and the cross-correlation for improving the detection accuracy can be easily implemented in the single radar by using this technique. The imbalance between the in-phase (*I*) and quadrature (*Q*) signals and a direct-current (DC) offset, which are critical characteristics for the accuracy of the phase measurement, are calibrated by modifying the method used by the CW Doppler radar sensor for the FSK radar. Distance measurements with the proposed radar indicate that the phase difference of vital signs at each operating frequency have a cross-correlation. The measurement results of heartbeat detection have high accuracy relative to the reference electrocardiogram (ECG) signal owing to the cross-correlation of the proposed FSK radar. The measurement results for the distance and heartbeat show that the detection accuracy at each frequency depends on the distance, owing to the characteristics of the FSK radar. The operating principles of the FSK radar for vital-signs detection are described in Section 2. Section 3 shows the implementation method of the proposed FSK radar, including the calibration process and digital signal processing. The measurement results for vital signs and distances and an analysis of the results discussed in Section 4. Conclusions are presented in Section 5. 

## 2. Vital-Signs Detection Using FSK Radar

### 2.1. Operating Principles for FSK Radar

**Figure 1:**
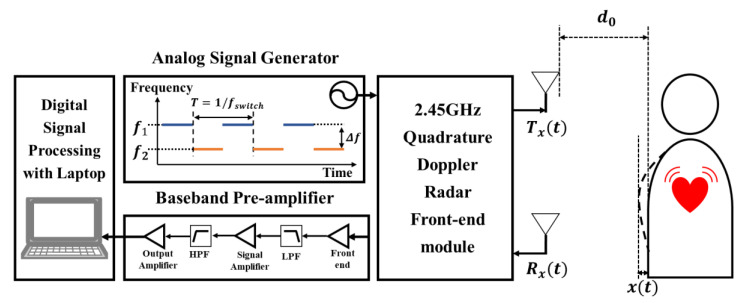
Block diagram of the proposed frequency-shift keying (FSK) radar.

An FSK radar measures the information of the target from the phase differences between two operating frequencies. The phase difference is obtained by comparing the transmitted and received signals at a single CW operating frequency. Accurate information can be obtained by increasing the measurement accuracy of the phase difference at each frequency. A block diagram of the FSK radar is shown in Figure 1. A quadrature architecture is adopted in the proposed FSK radar to avoid the null point problem in the CW Doppler radar [17,18]. Two discrete frequencies *f*_1_ and *f*_2_ are alternately transmitted for the switching time period of *T* with a duty cycle of 50% by a signal generator. A short time period is advantageous for increasing the measurement accuracy because the FSK radar assumes that the information of the target is constant during the period. However, a sufficient period for sampling baseband signals is needed to obtain the phase difference at each frequency. Thus, the time period should be optimized with consideration of the maximum velocity of the movement in the target and the maximum sampling frequency of the synchronous data-acquisition (DAQ) device.

The transmitted signals *T_x_*(*t*) in FSK radar can be expressed as follows:

(1) $$T_{x}\left(t\right)=\{\begin{array}{l} A_{T1}\cdot \cos\left[2\pi f_{1}t+\varphi_{1}\left(t\right)\right],\;0<t\leq \frac{T}{2}\\ A_{T2}\cdot \cos\left[2\pi f_{2}t+\varphi_{2}\left(t\right)\right],\;\frac{T}{2}<t\leq T \end{array},$$

where *A*_*T*1_ and *A*_*T*2_ represent the amplitudes of the transmitted signals, and *φ*_1_(*t*) and *φ*_2_(*t*) represent the phase noises of the two transmitted frequencies in the signal generator, respectively. The receiving signals in the radar are modulated to the Doppler frequencies produced by the chest movements caused by the respiration and heartbeat [18]. The received signals *R_x_*(*t*) in FSK radar can be expressed as follows:

(2) $$R_{x}\left(t\right)\approx \{\begin{array}{c} A_{R1}\cdot \cos\left[2\pi f_{1}t-\frac{4\pi d_{0}}{\lambda_{1}}-\frac{4\pi x_{1}\left(t\right)}{\lambda_{1}}+\varphi_{1}\left(t-\frac{2d_{0}}{c}\right)\right],\;0<t\leq \frac{T}{2}\\ A_{R2}\cdot \cos\left[2\pi f_{2}t-\frac{4\pi d_{0}}{\lambda_{2}}-\frac{4\pi x_{2}\left(t\right)}{\lambda_{2}}+\varphi_{2}\left(t-\frac{2d_{0}}{c}\right)\right],\;\frac{T}{2}<t\leq T \end{array},$$

where *A*_*R*1_ and *A*_*R*2_ represent the amplitudes of the received signals; *c* represents the velocity of light, *λ*_1_ and *λ*_2_ represent the wavelengths of the two frequencies, respectively; *d*_0_ represents the fixed distance between the radar and the target; and *x*_1_(*t*) and *x*_2_(*t*) represents the displacements of the chest caused by the respiration and the heartbeat. The phase noise of the received signal is described with the time delay of 2*d*_0_/*c* by considering the time of the round trip of the signal at the distance. The in-phase (*I*_1_ and *I*_2_) and quadrature (*Q*_1_ and *Q*_2_) baseband signals, which are obtained from the down-conversion quadrature mixers, can be expressed as follows:

(3) $$I_{k}\left(t\right)=A_{I}\cdot \cos\left[\frac{4\pi d_{0}}{\lambda_{k}}+\frac{4\pi x_{k}\left(t\right)}{\lambda_{k}}+\Delta \varphi_{k}\left(t\right)\right]+DCI_{k},\;k=1,2,\;\mathrm{and}$$

(4) $$Q_{k}\left(t\right)=A_{I}A_{E}\cdot \sin\left[\frac{4\pi d_{0}}{\lambda_{k}}+\frac{4\pi x_{k}\left(t\right)}{\lambda_{k}}+\Delta \varphi_{k}\left(t\right)+\phi_{E}\right]+DCQ_{k},\;k=1,2,$$

where *A_I_* represents the amplitude of the I-channel baseband signal; *A_E_* and *ϕ_E_* represent the errors of the amplitude and phase, respectively; *DCI_k_* and *DCQ_k_* represent the DC offset voltages in the *I* and *Q* channels, respectively; and Δ*φ_k_* represents the residual phase noise, which is the difference in the phase noise between the transmitted and received signals. The residual phase noise in the radar system can generally be neglected in the measurement of the distance and vital signs, owing to the range correlation effect [19]. The phase difference *θ_k_* between the transmitted and received signals at each frequency can be represented by using (3) and (4) as follows:

(5) $$\theta_{k}≅\frac{4\pi }{\lambda_{k}}\left(d_{0}+x_{k}\left(t\right)\right),\;k=1,2,$$

which is identical to the difference of the CW Doppler radar. The detectable range in the phase difference, which is 0–2*π*, is determined by the characteristics of the trigonometric function, as indicated by (5). The distance *d*_0_ can be measured from the subtraction in each phase difference using the two frequencies in the FSK radar, as follows:

(6) $$d_{0}=\frac{c}{4\pi \left(f_{1}-f_{2}\right)}\left(\theta_{1}-\theta_{2}\right)-\left[x_{1}\left(t\right)-x_{2}\left(t\right)\right].$$

If the difference in the vital signs generated during the transmitting and receiving signals of each frequency can be neglected, the absolute distance can be obtained as follows:

(7) $$d_{0}≅\frac{c}{4\pi \left(f_{1}-f_{2}\right)}\left(\theta_{1}-\theta_{2}\right).$$

Thus, the error of the distance measurement in the FSK radar can show the cross-correlation of the vital signs at each operating frequency, and a low error corresponds to a high correlation rate between two vital signals. The periodic signals in the baseband can represent the vital signs included in the phase difference, because the FSK radar can be regarded as a CW Doppler radar with independent single-frequency operation. When the human motion does not have periodicity or is located outside of the frequency band of the vital signs, the vital signs can be detected through fast-Fourier transform (FFT) if the receiver is not saturated by the motion. The measured vital signs in the frequency band can be expressed as follows:

(8) $$X\left(f\right)=\frac{\lambda_{k}}{4\pi }\int_{-\infty }^{\infty }\theta_{k}\left(t\right)e^{-j2\pi f_{m}t}dt.$$

### 2.2. Cross-Correlation Method

**Figure 2:**
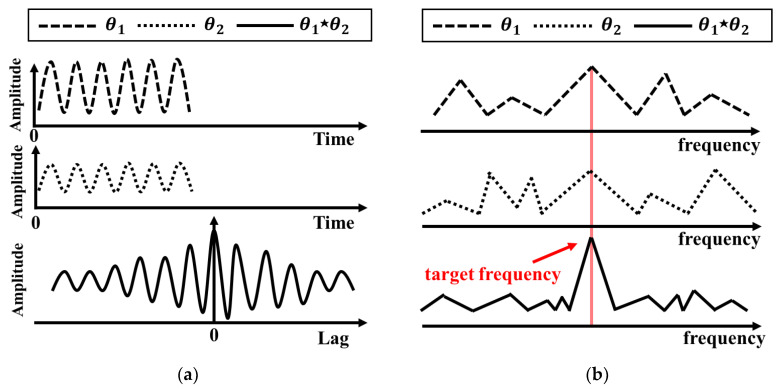
Cross-correlation method: (**a**) In the time domain; (**b**) in the frequency domain.

The cross-correlation method can be used for increasing the power of a periodic signal in a noisy environment [20]. Respiration and heartbeat, which are vital signs obtained directly from the CW radar, are both periodic signals, but the heartbeat of a stationary subject has a relatively low SNR compared with the respiration of the subject. In the proposed FSK radar, the cross-correlation method is used for accurately extracting the heartbeat from the raw data. The cross-correlation between the phase differences of signals at two operating frequencies can be mathematically expressed in the digital domain as follows:

(9) $${\hat{R}}_{\theta_{1}\theta_{2}}\left[m\right]=\overset{N-m-1}{\underset{n=0}{\sum }}\theta_{1}\left[n+m\right]\theta_{2}^{*}\left[n\right],\;m=1,\;2,\;\ldots ,\;2N-1,$$

where ${\hat{R}}_{\theta_{1}\theta_{2}}$ represents the result of the raw correlation between the *n*th element of *θ*_1_ and the *m*th element of *θ*_2_, and *N* represents the product of time and the sampling frequency [20]. The power levels of the elements simultaneously present in the two phase-different signals are increased using the cross-correlation method. The cross-correlated signal is expanded to double the length of the phase-difference signal in the time domain, as shown in Figure 2a. The frequency resolution of the signal is also increased in the frequency domain, as shown in Figure 2b, because the number of sampled data is increased at the same sampling frequency by the cross-correlation. Both white noise and noise signals caused by the hardware components that have different frequency characteristics are reduced by the cross-correlation. The proposed FSK radar can easily improve the vital-signs detection performance without additional signal processing, because the FSK radar uses the same hardware configuration to obtain the cross-correlated signal with the measured phase differences at the two operating frequencies. Thus, the peak detection accuracy of the heartbeat can be improved in the frequency domain because the SNR of the heartbeat in the proposed FSK radar is improved by the enhancement of the signal power level and the suppression of the noise level.

### 2.3. Frequency Discrimination Using Envelope Detection for Proposed FSK Radar

**Figure 3:**
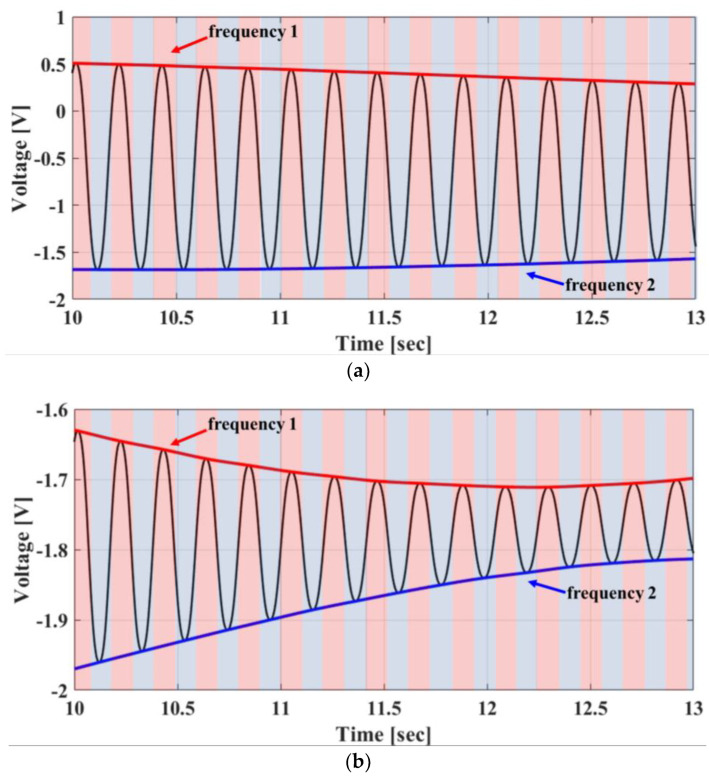
Proposed envelope detection method for discriminating the baseband signals at each operating frequency of the FSK radar. The frequency-control signal of the signal generator, which is indicated by red and blue shaders in the waveform, is used for synchronous data acquisition: (**a**) In-phase channel signals; (**b**) quadrature channel signals.

The discrimination of phase-difference signals at two different operating frequencies is the most important process for implementing the FSK radar. However, the signals at two frequencies are not continuous by the FSK operation, and the sampling points at each frequency are not identical. An envelope detection method can discriminate two nonoverlapped signals with different DC offsets at each frequency, because the offset characteristics of the radar components depend on the operating frequency [16]. This method is implemented using spline interpolation with not-a-knot conditions over local maxima separated by 10 samples. The baseband signals can be discriminated in each frequency by using the envelope detection method, but it cannot be known whether the discriminated signal shows anything of the two frequencies. The control signal for frequency switching in the signal generator is used to store each data-set of *I/Q* channel signals with each discriminated frequency in four data-sets. In the initial state, the stored data-set is regarded as the signals at *f*_1_ when the control signal is high, and the data-set is regarded as the signals at *f*_2_ when the control signal is low. The frequency is adjusted to ensure that the distance obtained from each data-set is positive. Figure 3a,b show the baseband *I/Q* signals for each frequency extracted from the raw data by the envelope detection method. 

## 3. Implementation

### 3.1. Digital Signal Processing

**Figure 4:**
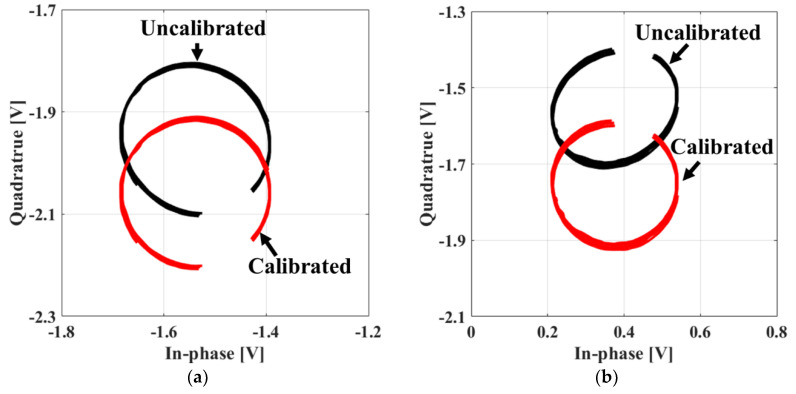
In-phase/quadrature (I/Q) imbalance calibration on the complex plane using the reference distance and the known periodic movement: (**a**) At the frequency of 2.45 GHz; (**b**) at the frequency of 2.5 GHz.

The detection accuracies of both the vital signs of the subject and the distance to the subject are determined by how accurately the phase difference is measured in the FSK radar, and they are significantly affected by the *I/Q* imbalance and DC offset in the baseband. The *I/Q* imbalance in the amplitude and phase caused by the imperfections of the quadrature receiver can be measured by phase shifters and calibrated by the Gram–Schmidt procedure [21]. The phase shifter is used to generate a circle trajectory by varying the phases of the signal on the complex plane, and several other methods can be employed to implement this function. When the target is mechanically moved within a displacement similar to the half wavelength of the operating frequency, baseband signals can draw the circle trajectory on the complex plane as in the case of using the phase shifter [22]. However, an elliptical trajectory is generated owing to the change in the distance to the target, in contrast to the circle trajectory using phase shifters. The elliptical trajectory can be compensated by the data-based quadrature imbalance compensation technique using the ellipse fitting method [23]. Figure 4a,b present the calibrated *I/Q* channels after the imbalance calibration. 

**Figure 5:**
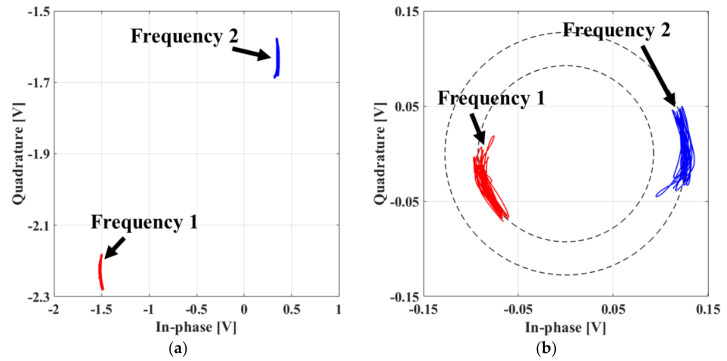
Direct-current (DC) offset calibration on the complex plane: (**a**) Uncalibrated signals of each frequency in the baseband; (**b**) Calibrated signals of each frequency based on the Pratt method.

DC offsets are generated by stationary clutters in the surroundings as well as the imperfection of the hardware configuration of the radar. The FSK radar receives the vector-sum signals reflected from all objects located in the antenna beamwidth [15]. The accuracy of the phase difference can decrease as the measurement distance increases because the DC offsets increase with the amount of clutters received on the radar. The DC offsets on the radar can be eliminated while preserving vital signs, which are located near DC in the frequency domain, using a dynamic DC offset compensation algorithm [24]. Figure 5 presents the baseband *I/Q* signals at each frequency measured by the proposed FSK radar on the complex plane. The trajectory at each frequency is a part of each circle on the complex plane, and the DC offset voltage is indicated by the center of the circle by the circle fitting method using the trajectory. The DC offset can be effectively calibrated by this procedure, which is known as the Pratt method, when the circle trajectory is obtained from the measured data [25].

**Figure 6:**
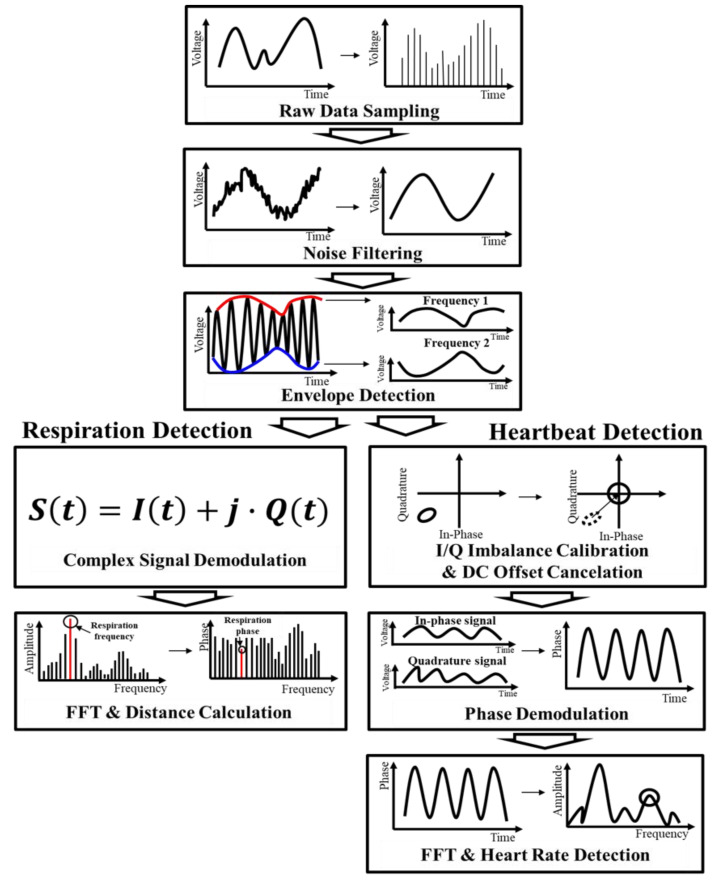
Signal processing procedure in the proposed vital-signs detector.

The phase difference in each frequency is extracted by demodulating the calibrated signals. In the proposed FSK radar, the arc-sine demodulation and the complex signal demodulation (CSD) techniques are used for detecting the vital signs of the subject and the distance to the subject, respectively [26,27]. The arc-sine demodulation technique is generally used as a demodulation technique, but is not suitable for distance measurement with the FSK radar, because the measurement error can significantly increase with the decreasing accuracy of the DC offset when accurate circle fitting cannot be realized via either the random body movement or the curved chest wall of the human body. The CSD technique is an appropriate demodulation method in distance measurement of the FSK radar, because an accurate circle fitting process is not mandatory in the CSD, in contrast to the arc-sine demodulation technique [28]. Bandpass filters with cutoff frequencies ranging from 0.8 to 2 Hz and from 0.1 to 0.8 Hz are used after the demodulation process to extract the respiration and heartbeat signals, respectively, in these two techniques. Considering the characteristics of the vital signs, which vary irregularly, FFT with a sliding window of 30 s is performed every 10 s with a measurement time of 90 s. Figure 6 shows the signal processing procedure to obtain the vital signs from the raw data of the proposed radar. 

### 3.2. Implemented FSK Radar Module

**Figure 7:**
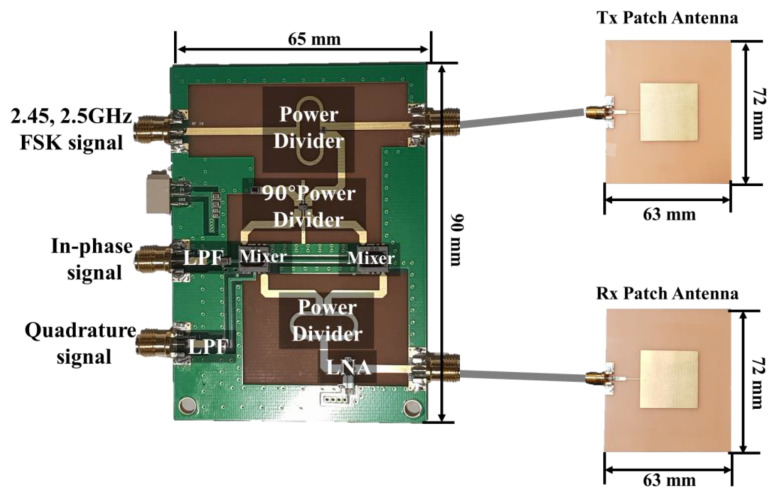
Implemented radar module with two patch antennas on an FR4 printed circuit board (PCB).

For vital-signs and distance measurement, the frequencies of 2.45 and 2.5 GHz are used in the proposed FSK radar, which operates in the 2.45 GHz ISM band. The maximum unambiguous range of the FSK radar is 3 m, which is determined by the frequency difference of 50 MHz. The radar front-end circuit and two patch antennas are implemented on an FR4 printed circuit board (PCB) with a thickness of 1 mm, as shown in Figure 7. The FSK signals with a frequency spacing of 50 MHz and an output power of 15 dBm are generated by an N5183B signal generator manufactured by Keysight Technologies Inc. The FSK operation is realized by using the internal function of the signal generator. The generated signals are divided by a Wilkinson power divider into the reference and transmitting signals. Quadrature signals are generated by a hybrid power divider with a phase difference of 90° between two outputs. The signal of each frequency is radiated toward the subject and received by the separated patch antennas with a directivity of 5.9 dBi. The received signal is amplified using a low-noise amplifier (LNA) with a power gain of 13.7 dB and noise figure of 5.3 dB. In-phase and quadrature signals in the baseband are generated by mixing the received signals with the reference and filtering them with low pass filters having a cut-off frequency of 80 MHz.

## 4. Measurement Results and Discussions

**Figure 8:**
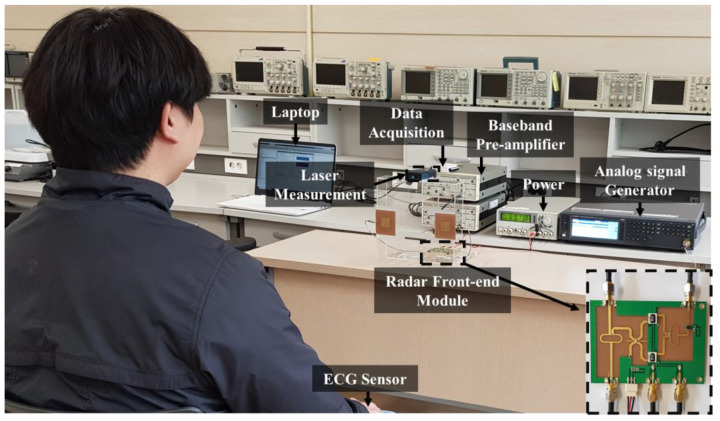
Measurement setup for detecting the distance and vital signs using the proposed FSK radar.

Figure 8 shows the measurement setup for obtaining both the vital signs and the distance to the subject. The switching time of the two CW frequencies was set as 0.1 s in the generator. The maximum unambiguity range was determined to be 3 m by the frequency space of 50 MHz between the two operating frequencies. By using two low-noise preamplifiers manufactured by Standford Research Systems Inc., the in-phase and quadrature signals from the module were amplified with a voltage gain of 17 dB. Signal conditioning and processing in the digital domain were implemented using NI LabVIEW and MATLAB on a personal computer after quadrature signals were simultaneously obtained with a sampling rate of 1 k samples per second from the DAQ board of NI USB-6009. A range finder (Bosch, Gerlingen, Germany) and a three-electrodes ECG sensor (Vernier Software and Technology, Beaverton, OR, USA.), were used as the reference sensors to measure the accuracies of the distance and vital signs, respectively, of the proposed FSK radar. In the surroundings, there were only fixed clutters; the moving objects (except for the subject) were limited in number. The distance to the subject was measured from 1 to 2.4 m at the intervals of 15 cm, and the vital signs were measured as respiration and heartbeat per minute for 90 s at each distance. The detection range for the demonstration is determined by considering both the nearfield effect on the radar and the maximum unambiguity range of the FSK radar. The subjects were three males in their twenties who did not suffer from cardiac diseases. They did not consume caffeine, alcohol, or nicotine, which may affect the vital signs, before measurement of the vital signs. Detailed information of the subjects is presented in Table 1.

**Figure 9:**
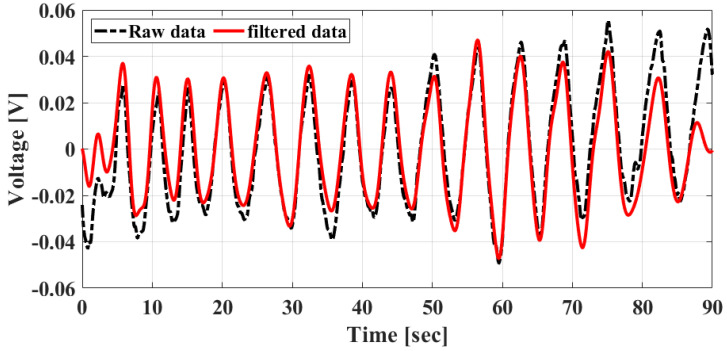
Raw and filtered waveforms measured at a distance of 1 m using the proposed radar.

The single-frequency in-phase signals obtained after the discrimination using the proposed envelope detection method and the signals digitally filtered by the passband from 0.1 to 0.8 Hz are shown in Figure 9. According to the operation of the CW Doppler radar, the phase information for the movement generated by the vital signs is clearly reflected by the raw data. As shown in Figure 9, the respiration is dominant in the raw data because the data are similar to the signal filtered by only the frequency band of the respiration.

**Figure 10:**
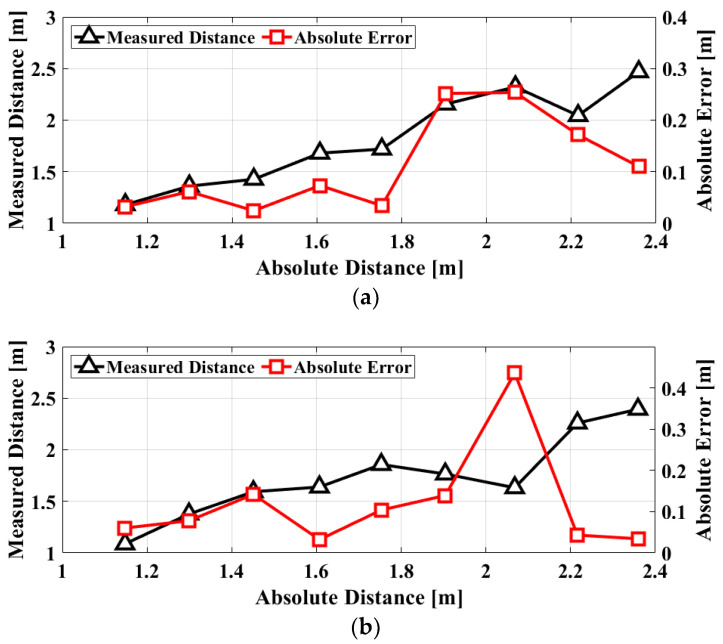

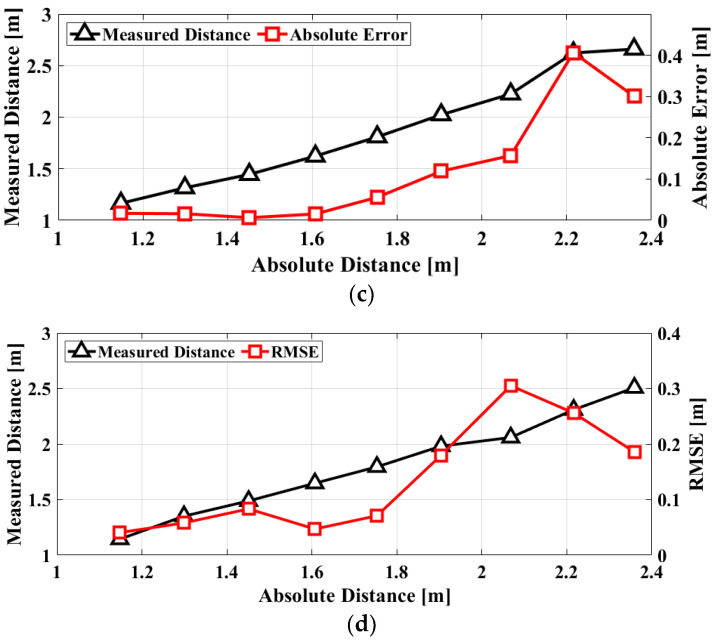
Measured distance and the root-mean-square error (*RMSE*) in the measurement using the proposed FSK radar with a frequency spacing of 50 MHz and switching time of 0.1 s: (**a**) Subject A; (**b**) Subject B; (**c**) Subject C; (**d**) averaged data.

The absolute distances measured by using the proposed FSK radar are shown in Figure 10. The initial phase differences in the radar front-end are calibrated with measurement results for the reference distance of 1 m, and the relative distance obtained by the radar is modified to an absolute distance after the calibration. The accuracy of the distance measurement is expressed by the root-mean-square error (*RMSE*) relative to the distance measured by the reference sensor. The *RMSE* in the distance measurement can be expressed as

(10) $$RMSE=\sqrt{\frac{1}{n}\overset{n}{\underset{i=1}{\sum }}{\left(d_{i}-rf_{i}\right)}^{2}}$$

where *n* is the number of subjects, *d_i_* is the absolute distance measured using the proposed radar, and *rf_i_* is the reference distance measured using the laser-based range finder. The *RMSE* is ≤0.1 m at a distance of ≤ 1.7 m, corresponding to ≤ 6.6% of the measurement distance. The accuracy is increased with an *RMSE* of 0.3 m at a distance of 1.8–2.4 m, which corresponds to 14.3% or less of the measurement distance. The increase in the measured distance error at the distances of >1.6 m is explained as follows: The phase difference of the FSK radar is determined by the vector sum of the overall signal received through the antenna. When the measurement distance increases, more information resulting from clutters is included in the received signal by the beamwidth of the receiving antenna, and it has an effect on the increase of the measurement error in the phase detection. Additionally, the SNR of the radar decreases as the measurement distance increases. The FSK radar measures the distance using the phase difference obtained from the vector sum of the overall reflected signals, including those from the surface and inside of the human body, while the reference sensor measures the absolute distance to the skin surface of the subject. The uncertainty of the phase difference obtained by the vector sum in the FSK radar results in an intrinsic error in the distance measurement for the human body. Additionally, the switching time of 0.1 s in the FSK radar is sufficiently short to neglect the changes in the vital signs, which occur less than 2 times per second. Considering the uncertainty of the phase difference and the short switching time, the error in the distance measurement of the radar is attributed to the detection accuracy of the phase in the radar. If the phase error of the FSK radar, including the uncertainty, is 5° in the measurement, the *RMSE* is 0.04 m for the frequency spacing. The distance measurement shows that vital signs obtained for each frequency at different times have a correlation at a similar level of the phase error in the measured distance.

**Figure 11:**
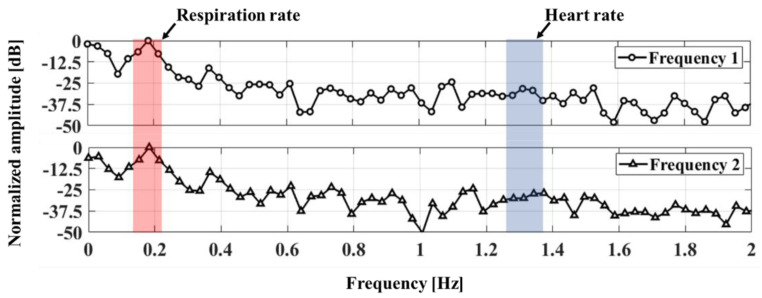
Spectra of the vital signals measured at a distance of 1 m using the proposed FSK radar.

**Figure 12:**
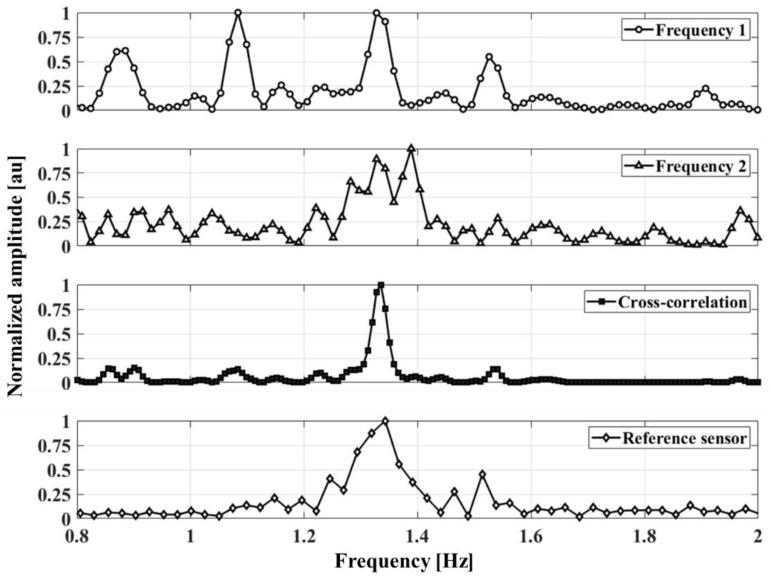
Spectra of the heartbeat signals measured by the proposed FSK radar at each operating frequency and with cross-correlation. The frequency peak of the cross-correlated signals is almost identical to that of the reference electrocardiogram (ECG) signals.

Vital-signs detection using the proposed radar are performed by varying the subjects’ position by 1 to 2.4 m at intervals of 0.15 m for a measurement time of 90 s. The respiration rate and heartbeat measured by the proposed radar are presented in the spectrum. The respiration rate per minute shown in Figure 11 for one subject is clearly measured at 12, but the heartbeat per minute (BPM) is not easily obtained in Figure 11 because of the low SNR of the heartbeat signal and the low resolution in the dynamic range to represent the respiration signal. The heartbeat signal may be measured by using high-pass filtering to reduce the respiration signal in the baseband pre-amplifier block, but this method has a limitation in increasing the measurement accuracy because it does not improve the heartbeat SNR itself. Figure 12 shows the frequency spectra of the heartbeat signals measured at a distance of 1 m using the proposed FSK radar. The raw data of the measured heartbeat show that signals due to the respiration and its harmonics might have been generated mostly around the frequency band of the heartbeat signal in the single CW radar operation. It is difficult to identify the frequency peak representing the heartbeat, because there are several peaks in the spectrum obtained at each operating frequency. The noise fluctuation level obtained at each frequency is approximately a quarter of the maximum frequency. However, the cross-correlated signals obtained using the proposed FSK radar exhibit the same frequency peak as those obtained using the reference ECG sensor and both higher SNR and lower noise level compared to those obtained at each frequency. The number of data points in Figure 10 (based on a comparison between the single frequency and cross-correlated signals) indicates that the frequency resolution can be increased by the cross-correlation. 

**Figure 13:**
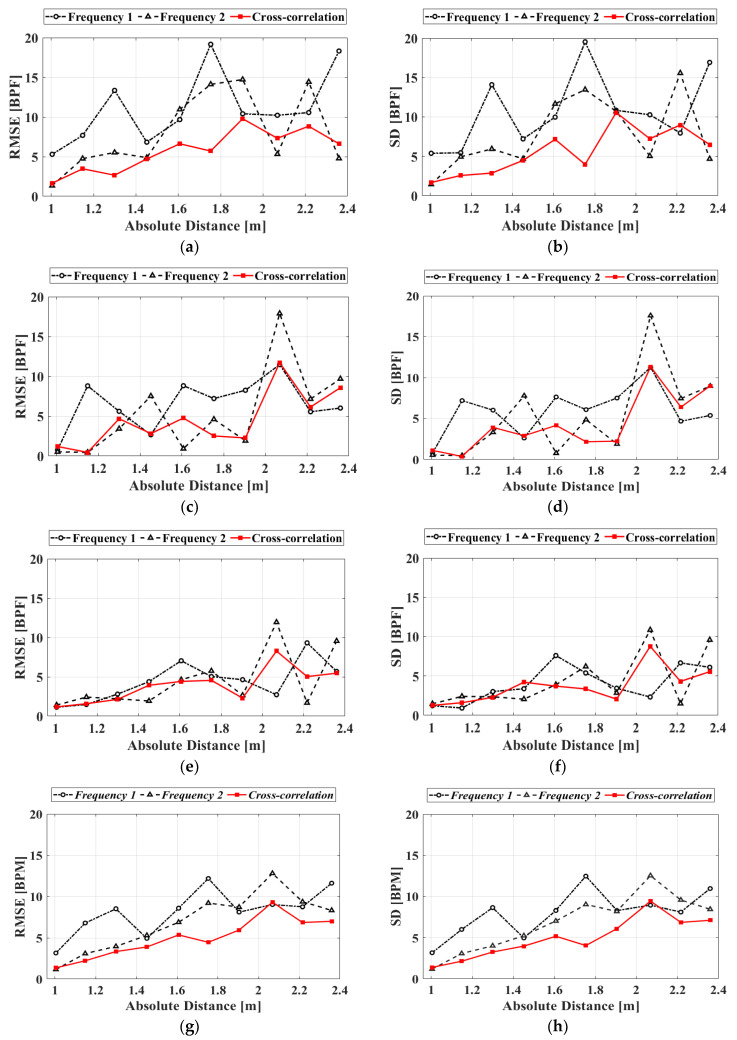
Measured accuracies of the signals obtained at each operating frequency and signals processed with the cross-correlation method in the proposed FSK radar depending on the distance to the subjects: (**a**) Root-mean-square error (*RMSE*) of subject A; (**b**) standard deviation (SD) of subject A; (**c**) *RMSE* of subject B; (**d**) SD of subject B; (**e**) *RMSE* of subject C; (**f**) SD of subject C; (**g**) *RMSE* of the averaged data; (**h**) SD of the averaged data.

The measurement accuracy for a subject wearing a contact-type reference ECG sensor is evaluated using the *RMSE* and the SD. The *RMSE* in the heartbeat measurement can be expressed as

(11) $$RMSE=\sqrt{\frac{1}{m}\overset{m}{\underset{i=1}{\sum }}{\left(h_{i}-ECG_{i}\right)}^{2}}$$

where *m* is the number of windows, *h_i_* is the heartbeat (BPM) measured using the proposed radar, and *ECG_i_* is the reference BPM measured using the commercialized ECG sensor. The BPM is measured for three subjects using the proposed FSK radar. The heartbeat measurement results in Figure 13 are the *RMSEs* and SDs of the subjects depending on the measurement distance. The results of the proposed FSK radar typically exhibit a smaller *RMSE* and SD than those measured at individual operating frequencies, i.e., those for the CW Doppler radar. When the measurement distance is increased, both the *RMSE* and SD of the heartbeat measurement increase corresponding to the reduction in the SNR. At a distance of 2.05 m, the accuracy obtained from the cross-correlated signals is similar to that obtained from the single-frequency signals. This indicates that the accuracy improvement due to the cross-correlation for the proposed FSK radar might not be significant when the effect of the low SNR and the uncertainty of the phase measurement increase owing to the increase in distance from the radar. Table 2 presents the average *RMSEs* and SDs for all BPM measurements for each subject. As shown, the cross-correlated signals had small *RMSEs* and SDs for all the subjects. In the proposed FSK radar, the average *RMSEs* and SDs of the heartbeat obtained using the cross-correlation method are improved by 2.42 and 2.36 BPM, respectively, compared to those measured at each frequency. With regard to the operating principle and the procedure, heartbeat measurement with only a single frequency using the proposed radar is identical to that using the CW Doppler radar. The measurement results demonstrate that the proposed FSK radar with the cross-correlation in a single hardware configuration is advantageous for improving the accuracy of vital-signs detection compared to the CW Doppler radar. Table 3 summarizes the performance comparison of the proposed radar with the radars from our previous studies, which were employed for vital-signs detection in similar measurement surroundings. 

## 5. Conclusions

A remote vital signs detector based on FSK radar with the cross-correlation method implemented in a single radar architecture is presented for accurately detecting heartbeat signals. The proposed FSK radar can increase the SNR of vital signs and the frequency resolution in the FFT spectrum by using cross-correlation between the phase differences individually obtained at two operating frequencies. An envelope detection method for controlling the frequency-switching signal for FSK operation is proposed for phase-difference discrimination, which is mandatory for obtaining vital signs at each operating frequency. Distance measurements for human subjects shows that the vital signs obtained at each operating frequency of the proposed FSK radar can be correlated with each other while producing an acceptable distance error. The measurement results for the number of heartbeats per minute for subjects at a distance of 1–2.4 m demonstrate that the proposed radar can improve the vital-signs detection accuracy and SNR via cross-correlation.

## Figures and Tables

**Table 1:** Information relevant to the subjects.

Subjects	Gender	Age	Weight	Height	BMI
A	Male	25	90	177	28.73
B	Male	28	76	182	22.94
C	Male	25	83	172	28.06

**Table 2:** Average root-mean-square error (*RMSE*) and standard deviation (SD) of overall heartbeat per minute measured for each subject by using the proposed FSK radar.

Subject	*RMSE* in BPM			SD in BPM		
	2.45 GHz	2.5 GHz	CC ^1^	2.45 GHz	2.5 GHz	CC ^1^
A	11.970	9.347	6.260	11.814	9.324	6.264
B	7.160	7.478	5.599	7.032	7.524	5.640
C	5.037	5.609	4.393	5.034	5.550	4.422
Average	8.562	7.632	5.472	8.550	7.602	5.460

^1^ The cross-correlation method.

**Table 3:** Performance comparison of the proposed radar with the radars from our previous studies, which were used for vital-signs detection in a similar measurement environment.

	[5]	[10]	[14]	This Work
**Type**	CW	CW	CW	FSK
**Operating Frequency (GHz)**	2.45	0.915	2.45	2.45 & 2.5
**Distance to target (m)**	0.4	0.2–1.4	1.0	1.0–2.4
**Signal Processing**	Peak detection	Fast-Fourier transform	Wavelet transform	Cross-correlation
**Detectable Information**	Respiration, heartbeat	Respiration, heartbeat	Respiration, heartbeat	Respiration, heartbeat, distance
**Mean absolute error in HR ^1^ (%)**	3.22	1.37	3.93	6.00
**Reference ECG sensor**	Three-electrodes sensor manufactured by Vernier Software and Technology			

^1^ Calculated by using the average error of heartrate (BPM) measured in all detectable ranges.

## References

[1] Lin J.C. (1975). Noninvasive microwave measurement of respiration. Proc. IEEE.

[2] Chen K.-M., Misra D., Wang H., Chuang H.-R., Postow E. (1986). An X-band microwave life-detection system. IEEE Trans. Biomed. Eng..

[3] Chen K.-M., Huang Y., Zhang J., Norman A. (2000). Microwave life-detection systems for searching human subjects under earthquake rubble or behind barrier. IEEE Trans. Biomed. Eng..

[4] Lin F., Zhuang Y., Song C., Wang A., Li Y., Gu C., Li C., Xu W. (2016). SleepSense: A noncontact and cost-effective sleep monitoring system. IEEE Trans. Biomed. Circuits Syst..

[5] Kim J.-Y., Park J.-H., Jang S.-Y., Yang J.-R. (2019). Peak detection algorithm for vital sign detection using Doppler radar sensors. Sensors.

[6] Brüster C., Antink C.H., Wartzek T. (2015). Ambient and unobtrusive cardiorespiratory monitoring techniques. IEEE Rev. Biomed. Eng..

[7] Shang N., Xin K., Yu C., Wang L. (2019). Design of direct wave cancellation system for high-frequency CW radar. J. Eng..

[8] De Groote A., Wantier M., Chéron G., Estenne M., Paiva M. (1997). Chest wall motion during tidal breathing. J. Appl. Physiol..

[9] Ramachandran G., Singh M. (1989). Three-dimensional reconstruction of cardiac displacement patterns on the chest wall during the P, QRS and T-segments of the ECG by laser speckle interferometry. Med. Biol. Eng. Comput..

[10] Park J.-H., Jeong Y.-J., Lee G.-E., Oh J.-T., Yang J.-R. (2019). 915-MHz continuous-wave Doppler radar sensor for detection of vital signs. Electronics.

[11] Guanghao S., Matsui T. Rapid and stable measurement of respiratory rate from Doppler radar signals using time domain autocorrelation model. Proceedings of the 2015 37th Annual Int. Conf. of the IEEE Eng. in Medicine and Biology Society.

[12] Li M., Lin J. (2018). Wavelet-transform-based data-length-variation technique for fast heart rate detection using 5.8-GHz CW Doppler radar. IEEE Trans. Microw. Theory Techn..

[13] Iyer B., Garg M., Pathak N.P., Ghosh D. Concurrent dual-band RF system for human respiration rate and heartbeat detection. Proceedings of the 2013 IEEE Conf. on Information & Communication Technologies.

[14] Choi C.-H., Park J.-H., Lee H.-N., Yang J.-R. (2019). Heartbeat detection using a Doppler radar sensor based on the scaling function of Wavelet transform. Microw. Opt. Techn. Lett..

[15] Yang J.-R., Hong S. (2014). A 24-GHz radar sensor with a six-port network for short-range detection. Microw. Opt. Techn. Lett..

[16] Park J.-H., Yang J.-R. (2020). Two-tone CW Doppler radar based on envelope detection method. Microw. Opt. Techn. Lett..

[17] Hu W., Zhao Z., Wang Y., Zhang H., Lin F. (2014). Noncontact accurate measurement of cardiopulmonary activity using a compact quadrature Doppler radar sensor. IEEE Trans. Biomed. Eng..

[18] Droitcour A.D., Boric-Lubecke O., Lubecke V.M., Lin J., Kovacs G.T. (2004). Range correlation and *I/Q* performance benefits in single-chip silicon Doppler radars for noncontact cardiopulmonary monitoring. IEEE Trans. Microw. Theory Techn..

[19] Siddiq K., Hobden M.K., Pennock S.R., Watson R.J. (2019). Phase noise in FMCW radar systems. IEEE Trans. Aerosp. Electron. Syst..

[20] Smith S. (2003). Digital Signal Processing: A Practical Guide for Engineers and Scientists.

[21] Wang J., Wang X., Chen L., Huangfu J., Li C., Ran L. (2013). Noncontact distance and amplitude-independent vibration measurement based on an extended DACM algorithm. IEEE Trans. Instrum. Meas..

[22] Yang J.-R., Kim D.-W., Hong S. (2007). A calibration method of a range finder with a six-port network. IEEE Microw. Wireless Compon. Lett..

[23] Singh A., Gao X., Yavari E., Zakrzewski M., Cao X., Lubecke V., Boric-Lubecke O. (2013). Data-based quadrature imbalance compensation for a CW Doppler radar system. IEEE Trans. Microw. Theory Techn..

[24] Li C., Zhao H., Xi F. (2015). Accurate DC offset calibration of Doppler radar via non-convex optimization. Electron. Lett..

[25] Pratt V. (1987). Direct least-squares fitting of algebraic surfaces. ACM SIGGRAPH Comput. Graph..

[26] Fan T., Ma C., Gu Z., Lv Q., Chen J., Ye D., Huangfu J., Sun Y., Li C., Ran L. (2016). Wireless hand gesture recognition based on continuous-wave Doppler radar sensors. IEEE Trans. Microw. Theory Techn..

[27] Li C., Lin J. Complex signal demodulation and random body movement cancellation techniques for non-contact vital sign detection. Proceedings of the 2008 IEEE Int. Microw. Symp..

[28] Wang J., Karp T., Muñoz-Ferreras J.M., Gómez-García R., Li C. (2019). A spectrum-efficient FSK radar technology for range tracking of both moving and stationary human subjects. IEEE Trans. Microw. Theory Techn..

